# Enhanced Autophagic Activity Improved the Root Growth and Nitrogen Utilization Ability of Apple Plants under Nitrogen Starvation

**DOI:** 10.3390/ijms22158085

**Published:** 2021-07-28

**Authors:** Liuqing Huo, Zijian Guo, Qi Wang, Li Cheng, Xin Jia, Ping Wang, Xiaoqing Gong, Cuiying Li, Fengwang Ma

**Affiliations:** 1 State Key Laboratory of Crop Stress Biology for Arid Areas/Shaanxi Key Laboratory of Apple, College of Horticulture, Northwest A&F University, Shaanxi 712100, China; liuqingsugar@163.com (L.H.); zhijian12138@163.com (Z.G.); 17863802742@163.com (Q.W.); li123cheng@163.com (L.C.); jiaxin0904@sina.com (X.J.); tingtlc007@gmail.com (P.W.); gongxq0103@nwsuaf.edu.cn (X.G.); 2 Collaborative Innovation Center for Efficient and Green Production of Agriculture in Mountainous Areas, College of Horticulture Science, Zhejiang Agriculture and Forestry University, Hangzhou 311300, China

**Keywords:** apple, autophagy, *MdATG10*, nitrogen assimilation, nitrogen starvation

## Abstract

Autophagy is a conserved degradation pathway for recycling damaged organelles and aberrant proteins, and its important roles in plant adaptation to nutrient starvation have been generally reported. Previous studies found that overexpression of autophagy-related (ATG) gene *MdATG10* enhanced the autophagic activity in apple roots and promoted their salt tolerance. The *MdATG10* expression was induced by nitrogen depletion condition in both leaves and roots of apple plants. This study aimed to investigate the differences in the growth and physiological status between wild type and *MdATG10*-overexpressing apple plants in response to nitrogen starvation. A hydroponic system containing different nitrogen levels was used. The study found that the reduction in growth and nitrogen concentrations in different tissues caused by nitrogen starvation was relieved by *MdATG10* overexpression. Further studies demonstrated the increased root growth and the higher nitrogen absorption and assimilation ability of transgenic plants. These characteristics contributed to the increased uptake of limited nitrogen nutrients by transgenic plants, which also reduced the starvation damage to the chloroplasts. Therefore, the *MdATG10*-overexpressing apple plants could maintain higher photosynthetic ability and possess better growth under nitrogen starvation stress.

## 1. Introduction

Nitrogen (N) is a fundamental macronutrient for plant growth and development because it is a key component of many cellular constituents, including nucleic acids, amino acids, proteins, cell walls, membranes, chlorophyll, and phytohormones [1,2]. Nitrogen deficiency impedes plant growth, reduces photosynthesis, hydrolyzes cell proteins, promotes plant senescence, and ultimately decreases plant productivity [3,4]. Under aerobic conditions, NO_3_^−^-N is the predominant form of nitrogen in the soil for absorption and utilization by plants [5,6]. However, given the great significance of nitrogen fertilizer application to plant production, nitrogen fertilizer was used in agriculture excessively and inappropriately, and subsequently, a series of problems, such as air pollution and water pollution, have cropped up [7,8]. Therefore, the research on absorption and utilization of nitrogen by plants under limited nitrogen supply conditions may be of great significance to rational fertilization.

Plant production is reflected by the progress of photosynthesis during its life, which requires a system comprising many proteins [9]. Serving as a nitrogen storage unit, more than 70% of nitrogen in plants is stored in chloroplasts under normal conditions [10]. It is this large amount of nitrogen requirement to construct the photosynthetic system that results in the need for nitrogenous fertilizer. Most of the nitrogen in plants is transported to vigorous tissues and storage organs in response to nitrogen-deficient conditions [11]. Therefore, the loss of nitrogen from chloroplasts directly affects chlorophyll synthesis and leads to leaf senescence [12]. As leaves are the main organ for performing photosynthesis in plants, chlorophyll degradation and the leaf senescence phenotype lead to decreased photosynthetic capacity of plants [13].

The morphology and physiology of plant roots are affected by the nitrogen content in the soil, and the root structure, in turn, influences the nutrient and moisture absorption by plants under stress conditions [14]. The root length, distribution, and biomass are closely related to nitrogen uptake and utilization in response to nitrogen-deficient condition [15]. In addition, the root system is also an important site for assimilation and transformation of phytohormones and organic acids [16]. Nitrogen is taken up from soil by plants through energy expenditure and transport via transpiration. Research showed that NO_3_^−^-N was the primary form of nitrogen for absorption and utilization by plants [6]. After absorbing NO_3_^−^-N, most of it is transferred through the xylem to the aboveground mesophyll cells for reduction [17]. This reduction process first involves the reduction of nitrate to nitrite by nitrate reductase (NR) in the cytoplasm, and then nitrite is converted into ammonium by nitrite reductase (NiR) in plastids or chloroplasts. Ammonium is converted and assimilated as glutamic acid and glutamine through the action of glutamine synthetase (GS) and glutamate synthase (GOGAT) [18,19]. Among the absorption, assimilation, and transport of NO_3_^−^-N, the assimilation process is the most critical step and one of the most important limiting factors because both nitrite and excess ammonium are poisonous to plants [20]. Therefore, these assimilation steps need to be well coordinated at the cellular level in response to different nitrogen supply conditions, thereby promoting the nitrogen use efficiency of plants.

Autophagy plays an important role in nitrogen recycling when plants encounter starvation conditions [21,22]. The growth of various *Arabidopsis* autophagy-related (ATG) genes mutants is hypersensitive to nitrogen-deficient conditions by displaying early leaf senescence, lower rosette biomass, and reduced seed yield [23,24]. For example, both the *ATG18*-defective RNAi line and *atg5* mutants showed impaired autophagic activity and reduced nitrogen recycling ability compared with wild type (WT) *Arabidopsis* [25]. Similar results were found in crops, such as *Osatg7-1* mutant in rice, which showed reduced biomass production during the vegetative growth stage combined with suppressed nitrogen-remobilization in aging leaves [26]. The multiple omics studies on maize *atg12* seedlings showed that autophagy exerted significant effects on protein formation and membrane structure under nutrient-stress conditions [27]. A previous study on apple plants found that the levels of free amino acids were less reduced in *MdATG9*-overexpressing apple callus compared with the wild type under limited nitrogen conditions [28]. In addition, the research on *MdATG18a*-overexpressing apple plants showed that more anthocyanin was accumulated in the transgenic plants in response to nitrogen-deficiency stress, possibly due to the enhanced autophagic activity [29].

The research of *Arabidopsis* ATG10, the E2-like enzyme predicted to be responsible for ATG12 conjugation, showed that *atg10* mutants fail to form the ATG12-ATG5 conjugate and cannot accumulate autophagic bodies within the vacuole under nutrient deficiency condition [30]. A previous study in apple demonstrated that *MdATG10* had a conserved function in the apple salt tolerance [31]. As the present study found that the *MdATG10* expression could also be induced by low levels of nitrogen in both the leaves and roots of apple plants, here, we employed the transgenic and WT apple plants to investigate the effect of autophagy on the overall physiological and growth state of apple plants under nitrogen starvation. Through a series of studies, we found that *MdATG10* overexpression led to better root growth and more efficient absorption and assimilation of the limited nitrogen sources. Furthermore, this effective absorption of nutrition also reduced the starvation damage to chloroplasts in transgenic plants.

## 2. Results

### 2.1. Overexpression of MdATG10 Alleviated the Growth Limitation of Apple Plants under Nitrogen Deficiency Stress

**Figure 1:**
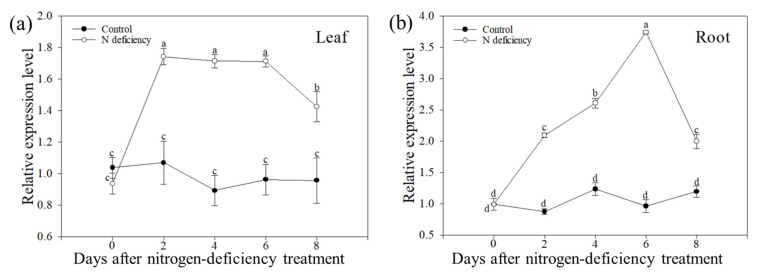
Changes in the expression of *MdATG10* in (**a**) leaf and (**b**) root of apple plants after treating with or without nitrogen depletion stress for 8 d. Different letters indicate significant differences between treatments, according to one-way ANOVA followed by Tukey’s multiple range test (*p* < 0.05).

The expression analysis of *MdATG10* under low-nitrogen stress showed that the *MdATG10* transcript was induced in both the leaves and roots, with respective upregulation being almost 1.8- and 3.8-fold on day 6 (Figure 1). Furthermore, to ensure the reliability of the conclusions of research, GL-3 (WT) and three previously obtained *MdATG10*-overexpressing (OE) apple lines (OE–1, OE–4, and OE–5) were used to further investigate the function of *MdATG10* in apple plants when they encountered nitrogen starvation conditions.

**Figure 2:**
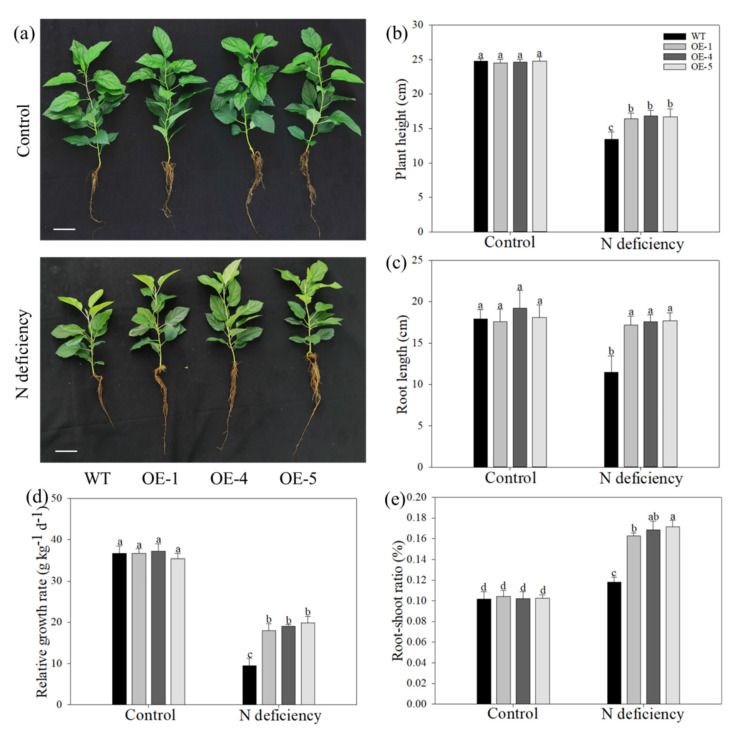
Overexpression of *MdATG10* alleviated the growth limitation of apple plants under nitrogen-deficiency stress. (**a**) Phenotypes of WT and transgenic apple plants under control hydroponic conditions and after 28 d of treatment with 0.15 mM nitrogen-deficiency stress. Bars: 5 cm. (**b**) Plant height, (**c**) root length, (**d**) relative growth rate, and (**e**) root–shoot ratio of WT and transgenic plants with or without treatment. Data are shown as the means of six replicates with SEs. Different letters indicate significant differences between treatments, according to one-way ANOVA followed by Tukey’s multiple range test (*p* < 0.05).

Using a hydroponics system, the WT and transgenic apple plants were cultured under control and low-nitrogen conditions for 28 days. The growth status was consistent between the OE and WT plants under control hydroponic conditions. After nitrogen starvation treatment, all genotypes exposed leaves chlorosis and growth inhibition, but the inhibition of transgenic plants was affected to a less serious extent than that of WT plants (Figure 2a and Table 1). The plant height decreased among the genotypes, but the reduction was much smaller in OE plants than in WT plants (Figure 2b). The same tendency was also found in the total FW or DW of OE and WT plants after nitrogen starvation treatment (Figure 2d,e). As for the root length, the limitation of root elongation caused by nitrogen deficiency was significant in WT plants, but this limitation was lessened by *MdATG10* overexpression (Figure 2c). The relative growth rate of the WT plants decreased to 25.9% of the control after treatment, while those of the OE plants decreased to 49.1% in OE–1, 51.0% in OE–4, and 56.1% in OE–5. Interestingly, the better root growth of transgenic plants caused the root–shoot ratio of them was significantly elevated under low-nitrogen conditions. These results suggested that the adverse effect of nitrogen deficiency on the growth of apple plants was effectively relieved by *MdATG10* overexpression.

### 2.2. Overexpression of MdATG10 Alleviated the Damage on the Photosynthetic System in Apple Plants under Nitrogen Deficiency Stress

**Figure 3:**
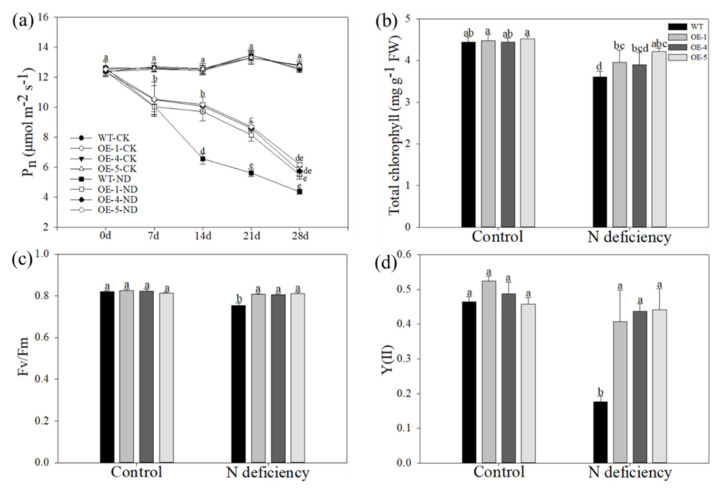
Overexpression of *MdATG10* leads to higher photosynthetic capacity in apple under nitrogen-deficiency stress. (**a**) Changes in the net photosynthesis rate (Pn) were determined every seven days during the treatment. (**b**) Total chlorophyll, (**c**) *Fv/Fm*, and (**d**) Y(II) of WT and transgenic plants with or without treatment. Data are shown as the means of three replicates with SEs. Different letters indicate significant differences between treatments, according to one-way ANOVA followed by Tukey’s multiple range test (*p* < 0.05).

Low levels of nitrogen had a negative effect on the plant’s photosynthetic system. Therefore, continuous measurements of net photosynthesis of all lines were performed during the nitrogen deficiency treatment. Under control conditions, the photosynthesis rate (Pn) was relatively consistent between the WT and transgenic plants as it was measured every 7 days. The nitrogen-deficiency condition led to the decrement in Pn of all apple plants, but the reduction was much less in the transgenic plants. In detail, while it declined drastically after 14 days of treatment in the WT plants, it was not apparently reduced in the OE plants until day 21 of treatment (Figure 3a). In addition, the detection of total chlorophyll content in the leaves of all apple plants after the treatment showed that the degradation of chlorophyll concentrations caused by low levels of nitrogen was much smaller in OE plants than in WT plants (Figure 3b). The same trend was also found in the changes in photosystem II (PSII) photochemistry between the WT and transgenic plants, as shown by the *Fv/Fm* (Figure 3c) and Y(II) (Figure 3d), which were measured on day 28 of treatment. These results suggested that the photosynthetic system of *MdATG10*-overexpressing apple plants was less damaged by nitrogen-deficiency stress than that of WT plants.

### 2.3. Apple Plants Overexpressing MdATG10 Maintained Better Growth and Activity of the Root System under Nitrogen-Deficiency Stress

**Figure 4:**
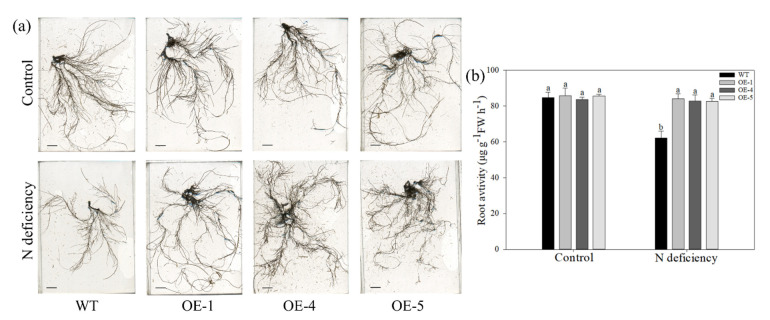
Overexpression of *MdATG10* leads to better root growth and activity in apple under nitrogen deficiency stress. (**a**) Root architecture, bars: 1 cm, and (**b**) root activity of WT and transgenic plants with or without treatment. Data are shown as the means of five replicates with SEs. Different letters indicate significant differences between treatments, according to one-way ANOVA followed by Tukey’s multiple range test (*p* < 0.05).

A significant difference in root biomass was observed between the OE and WT plants after nitrogen-deficiency treatment. Therefore, the root architecture of all genotypes was observed and analyzed (Figure 4a). Obviously, the root growth of WT plants was suppressed by a long-term low nitrogen level, but this suppression was not apparent in OE plants. Then, the root lengths, root volumes, surface area, and numbers of forks among genotypes were examined. For WT plants, these parameters were decreased by 44.3%, 45.0%, 41.3%, and 32.9%, respectively, at the end of the treatment (Table 2). However, for OE plants, the root lengths were decreased after treatment compared with the lengths of plants cultured under the control condition, but the other three indices of roots were not affected by nitrogen-deficiency stress. Moreover, the average root diameters did not differ among genotypes and treatment groups. Then, the root activity of all genotypes was measured. Only the root activity of WT plants was decreased by nitrogen deficiency; the OE plants were not significantly affected after treatment (Figure 4b). These results suggested that the restriction of both activity and growth of the transgenic plant root system caused by nitrogen deficiency was definitely reduced by *MdATG10* overexpression.

### 2.4. Apple Plants Overexpressing MdATG10 Maintained a Better Nitrogen Retention Ability under Nitrogen Deficiency Stress

**Figure 5:**
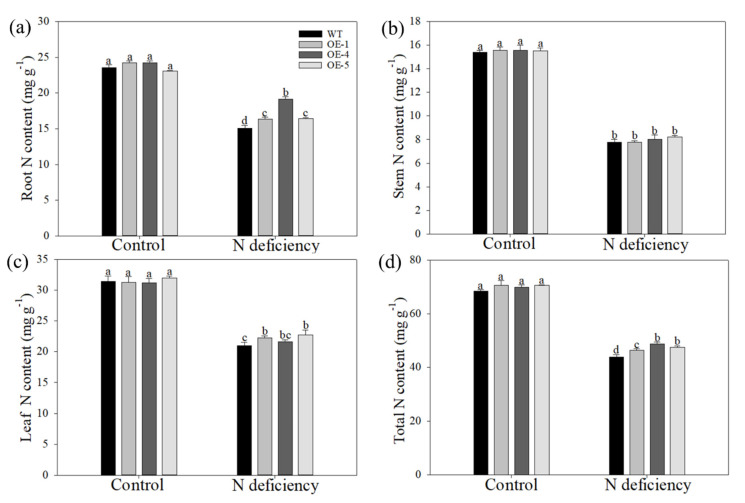
Overexpression of *MdATG10* leads to higher nitrogen concentration in apple under nitrogen deficiency stress. Changes of nitrogen concentrations in the (**a**) roots, (**b**) stems, (**c**) leaves, and (**d**) total of WT and transgenic apple plants. Data are shown as the means of three replicates with SEs. Different letters indicate significant differences between treatments, according to one-way ANOVA followed by Tukey’s multiple range test (*p* < 0.05).

Nitrogen is one of the most important mineral nutrients for plant growth and development. Furthermore, the autophagic recycling process in plants contributes to nitrogen remobilization under a nutrient-deficient condition. Under control hydroponics conditions, the measurement of nitrogen concentrations in the roots, stems, and leaves of apple plants showed no distinct differences among genotypes. The nitrogen starvation treatment greatly decreased the nitrogen content in different tissues, but the reduction was lower in the OE plants than in the WT plants (Figure 5a–d). For example, the nitrogen content in the roots of the WT plants decreased to 63.9% of the control after treatment, while those of the OE plants decreased to 67.4% in OE–1, 79.1% in OE–4, and 71.1% in OE–5 (Figure 5a).

**Figure 6:**
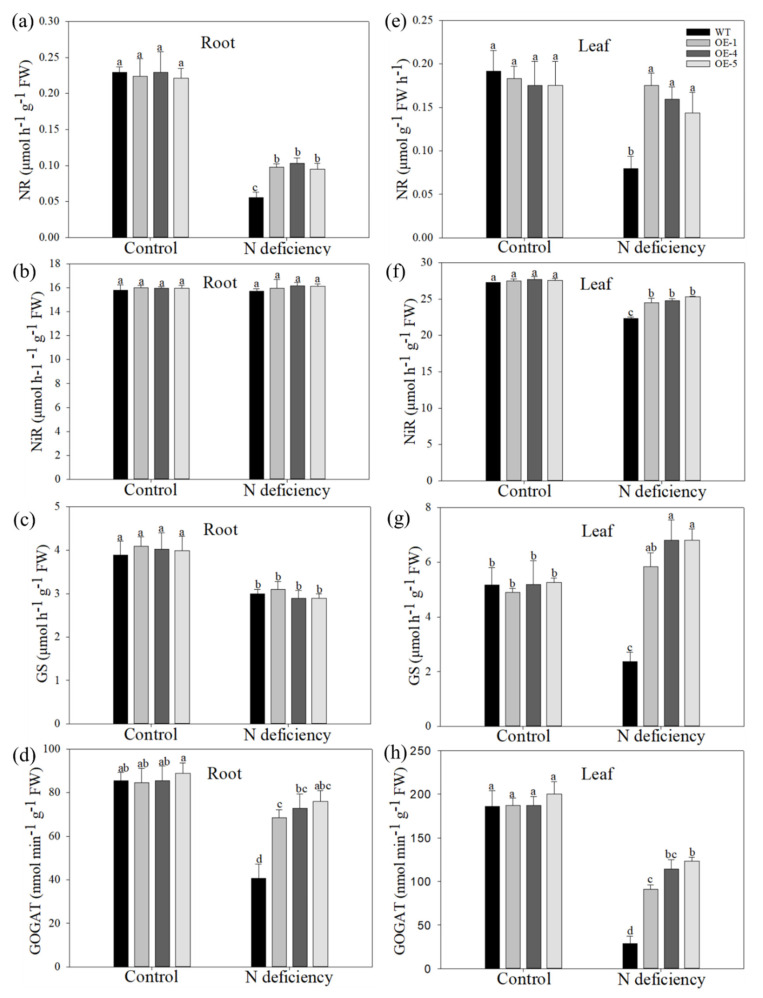
Overexpression of *MdATG10* leads to enhanced nitrogen assimilation ability in apple under nitrogen deficiency stress. Activities of (**a**) nitrate reductase (NR), (**b**) nitrite reductase (NiR), (**c**) glutamine synthetase (GS), and (**d**) glutamic acid synthase (GOGAT) in the root (**a**–**d**) and leaf (**e**–**h**) of WT and transgenic plants with or without treatment. Data are shown as the means of three replicates with SEs. Different letters indicate significant differences between treatments, according to one-way ANOVA followed by Tukey’s multiple range test (*p* < 0.05).

As NR, NiR, GS, and GOGAT enzymes play important roles in nitrate reduction and nitrogen assimilation in plants, the activities of these four enzymes in both the roots and leaves of transgenic apple plants and WT were measured. In roots, NR, GS, and GOGAT activities were decreased by nitrogen deficiency in all genotypes, but the activities of NR and GOGAT were significantly higher in OE plants than in WT plants after treatment (Figure 6a–d). In leaves, the difference in the activities of these four enzymes between WT and transgenic apple plants under treatment was even more pronounced. For example, the GS activity was diminished in WT plants but increased in the OE plants after treatment (Figure 6e–h). These results suggested that overexpression of *MdATG10* in apple plants improved its nitrogen absorption and assimilation ability under nitrogen-deficiency conditions.

### 2.5. Overexpression of MdATG10 Promoted the Expression of Nitrogen Absorption Genes in Apple Leaves under Nitrogen Deficiency Stress

**Figure 7:**
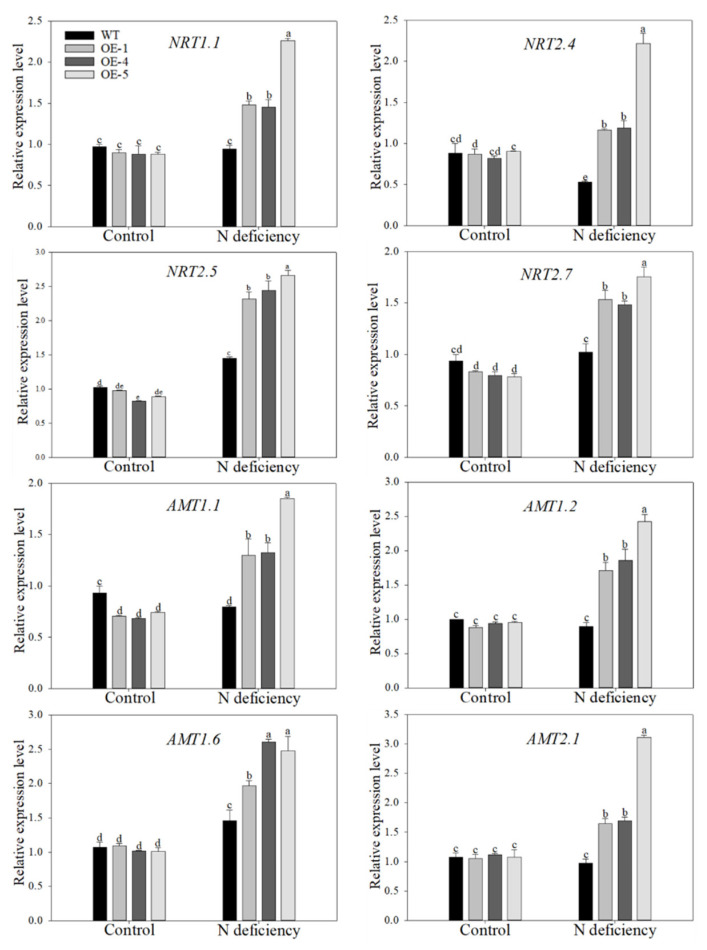
Changes in transcript levels of genes involved in nitrogen absorption in apple following nitrogen-deficiency treatment. Data are shown as the means of three replicates with SEs. Different letters indicate significant differences between treatments, according to one-way ANOVA followed by Tukey’s multiple range test (*p* < 0.05).

Due to the significant difference in the activities of enzymes involved in nitrogen assimilation pathways of transgenic and WT apple plants under low-nitrogen treatment, the expression of genes involved in the nitrogen absorption processes was examined in the leaves of all genotypes. As shown in Figure 7, the expression levels of four genes in the nitrate transporter (NRT) pathway, that is, *NRT1.1*, *NRT2.4*, *NRT2.5*, and *NRT2.7*, were all higher in the transgenic plants than in WT plants after 14 days of nitrogen-deficiency treatment. Particularly, while the expression of *NRT2.4* transcripts decreased in the WT plants under treatment, it was upregulated in the OE plants, even to almost threefold in OE–5. In addition, the expression pattern of ammonium transporter (AMT) genes showed the same trend as *NRT* genes among genotypes under treatment. For example, the expression of *AMT1.1* was downregulated by low levels of nitrogen in WT plants, but it was upregulated almost twofold in transgenic plants. The transcripts of *AMT*1.2, *AMT*1.6, and *AMT*2.1 were all expressed at higher levels in OE plants in response to nitrogen-starvation condition. These data demonstrated that overexpression of *MdATG10* upregulated some genes responsible for nitrogen absorption in the apple plants under nitrogen-deficiency conditions.

### 2.6. Overexpression of MdATG10 Intensified the Autophagic Activity in Apple Leaves under Nitrogen Deficiency Stress

**Figure 8:**
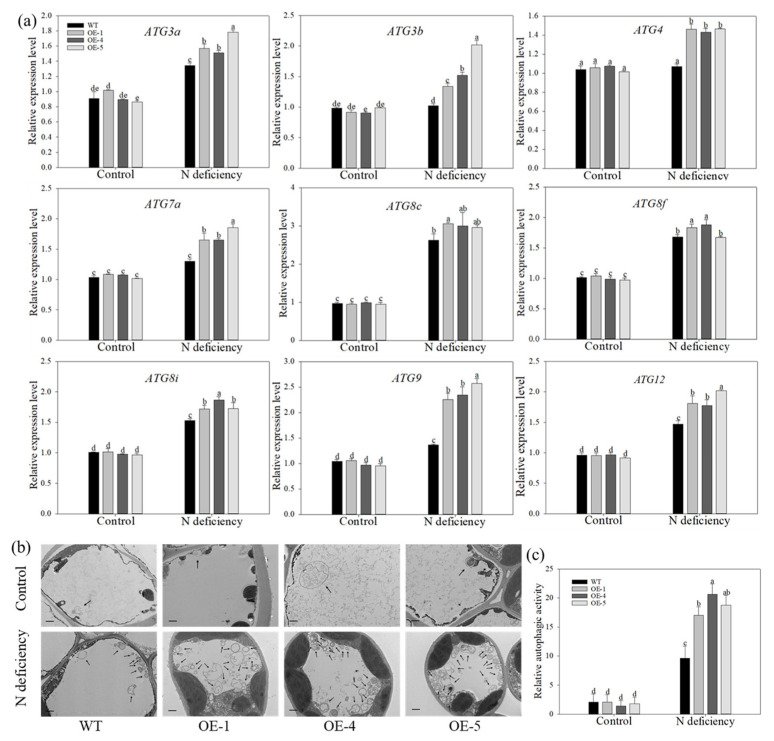
Expression of other *MdATGs* and formation of autophagosomes in apple following nitrogen deficiency treatment. (**a**) Changes in expression of other *MdATGs* in WT and *MdATG10*-OE plants following nitrogen deficiency treatment. (**b**) Representative TEM images of autophagic structures in mesophyll cells of WT and *MdATG10*-OE plants. Autophagic bodies are indicated by arrows. Bars: 1 μm. (**c**) Relative autophagic activity normalized to the activity of WT or *MdATG10*-OE plants shown in panel (**b**). More than 10 cells were used to quantify structures. Data are the means of six replicates with SE. Different letters indicate significant differences between treatments, according to one-way ANOVA followed by Tukey’s multiple range test (*p* < 0.05).

The autophagic recycling process plays an important role in plant resistance to a nutrient-deficient condition. qRT-PCR was used to examine the expression patterns of several important autophagy-related genes in the leaves of all plants to assess the changes in autophagic activity among plants with different genotypes under nitrogen deficiency treatment. The expression of detected *MdATGs* showed little difference among different genotypes under control hydroponics conditions. In response to low-nitrogen treatment, the expression of *MdATG3b* and *MdATG4* remained unchanged in WT plants, whereas the expression of *MdATG3a*, *MdATG7a*, *MdATG8c*, *MdATG8f*, *MdATG8i*, *MdATG9*, and *MdATG12* was induced among genotypes; all of them expressed at higher levels in the OE plants (Figure 8a). The changes in autophagic activity among different genotypes were further assessed under low-nitrogen treatment using a TEM. Under control hydroponic conditions, few autophagosome structures were observed in all the plants (Figure 8b). Increased numbers of autophagosomes were detected in apple plants in response to nitrogen starvation condition. The accumulation of autophagosomes was up to 4.7-fold in WT plants, but the accumulation in the OE plants increased to 8.2-fold in OE–1, 14.7-fold in OE–4, and 10.4-fold in OE–5 (Figure 8c). Taken together, the results indicated that the autophagy in apple plants triggered by low levels of nitrogen was intensified by *MdATG10* overexpression.

## 3. Discussion

Plants encounter a variety of physiological and biochemical changes during their growth. Therefore, they have developed many evolutionarily conserved strategies for stress resistance. Autophagy is one of the significant pathways for plants to maintain cell homeostasis in response to abiotic stresses, as many damaged organelles and redundant macromolecules are degraded through it [32,33]. Several studies demonstrated that autophagy could be induced by nutrient scarcity in different plant species, such as *Arabidopsis* [34] foxtail millet [35], rice [26], maize [36], and apple [28]. By placing the tissue-cultured *MdATG18a*-overexpressing apple plants on nitrogen-depleted MS medium, a previous study demonstrated that increased autophagy in apple plants led to enhanced tolerance to nitrogen deficiencies accompanied by increased anthocyanin accumulation in apple roots [29]. However, the effects of autophagy on the growth and physiological performance of apple plants under nitrogen-deficient conditions have not been systematically studied.

The present study found that *MdATG10* expression could be induced by nitrogen-starvation condition in the leaves and roots of apple plants. Three *MdATG10*-overexpressing apple plants were generated previously [30]. A hydroponic system was used to analyze the influence of enhanced autophagic activity on the growth and activity of apple plants under long-term low-nitrogen treatment. Under the hydroponic low-nitrogen treatment, the apple plants showed leaves chlorosis and growth inhibition, which was significantly alleviated by *MdATG10* overexpression. As nitrogen is the main component of chloroplasts, the external nitrogen level supplied to plants greatly affects the activity of enzymes in the photosynthetic system of leaves, thereby influencing the photosynthesis and production of plants [37,38]. The present study found that both the net photosynthesis and PSII photochemistry activity were higher in transgenic plants than in WT plants during the low-nitrogen treatment. Previous studies reported the active role of autophagy in nutrient remobilization when plants encountered starvation conditions [23,39]. It was believed that overexpression of *MdATG10* in apple plants could reduce the damage on chloroplasts under nitrogen deficiency stress by improving the recycling and utilization of limited nitrogen, which might be part of the reason for the differences in aboveground biomass among genotypes.

In addition, the long-term deficiency of nitrogen weakened the root growth of WT plants, but this suppression was not apparent in OE plants. The examination of root activity also showed the same tendency among genotypes. Particularly, the root–shoot ratio of transgenic plants increased under low-nitrogen conditions and was higher than that of WT plants. A previous study showed that the higher root–shoot ratio of maize made it conducive to survive under nitrogen deficiencies [15]. Here, the results demonstrated that *MdATG10* overexpression could reduce the restriction of root growth and further promote the root–shoot ratio of apple plants under nitrogen starvation conditions. The root system was the first portion of plants to perceive changes in the environmental nutrient concentrations; it was also the major organ responsible for absorbing external nutrients [40]. We believed that the better root system of *MdATG10*-overexpressing apple plants under low-nitrogen treatment could promote their uptake of limited nitrogen and other useful nutrients in hydroponics, which created a virtuous cycle for plant growth and might be another reason for the differences in biomass among genotypes.

For most plants, especially agricultural crops, the nitrate in soil was taken as the main nitrogen source [41]. The acquired inorganic nitrogen is absorbed through roots and reduced to amino acids in roots and leaves of plants [42]. In this study, the measurement of nitrogen concentration in different tissues showed that the nitrogen concentration was higher in the roots and leaves of transgenic plants than in WT after treatment. NR and NiR participate in the coupled regulation process of reducing nitrate to ammonium, and the activity of NR is mainly affected by the concentration of nitrate [43]. This study found that the NR activity was decreased by low-nitrogen treatment in both roots and leaves of WT plants, but it was significantly higher in OE plants than in WT in both tissues. The ammonium was assimilated as amino acids through the action of GS and GOGAT enzymes in the leaves of higher plants, and thus the external nitrate concentrations affected GS and GOGAT activities by influencing the reduction of ammonium [19]. This study found no significant differences in GS and GOGAT activities among genotypes under control conditions; however, the activities of these two enzymes were two to three times higher in the leaves of transgenic plants than those of WT plants under low-nitrogen treatment. Moreover, as only NO_3_^−^-N was supplied to apple plants in this research, accompanied by the fact that most of NO_3_^−^-N absorbed by plant roots is transferred to the aboveground mesophyll cells for reduction, the difference in the activities of NiR and GS was only found in the leaves of WT and OE plants in response to low-nitrogen treatment, but not in the roots. These results showed that the nitrate assimilation and utilization in transgenic plants, especially in the leaves, was less inhibited by nitrogen starvation conditions. Therefore, we believed that *MdATG10* overexpression could effectively curb the negative effect of external nitrogen deficiency on the endogenous nitrogen cycling in apple leaves, which increased the nitrogen utilization efficiency of apple plants.

In plants, the fluxes of nitrate and ammonium are mediated by various NRTs and AMTs [44]. The genetic approaches to improve the nitrogen use efficiency are associated with the manipulation of genes involved in inorganic nitrogen uptake and allocation [45] For example, the constitutive overexpression of the high-affinity nitrate transporters *OsNRT1.1b* in rice plants resulted in increased nitrate uptake and aboveground plant biomass grain yield [46]. Moreover, rice plants overexpressing *AMT1.1*, which is the most widely studied *AMT* gene in rice, showed superior growth and higher yield [47]. However, the researches on transporters involved in unloading and importing nitrogen in leaves and mesophyll are relatively scarce. *AtAMT1.1* and *AtAMT2.1* were identified for retrieving and importing ammonium into mesophyll cells in *Arabidopsis* [48,49]. Here, the changes in the expression patterns of key genes implicated in nitrogen uptake processes were examined in all genotypes. *MdATG10* overexpression upregulated the expression of most detected genes responsible for nitrogen absorption in apple plants under nitrogen-deficiency conditions. These results further proved the higher nitrogen absorption and utilization abilities of *MdATG10*-overexpressing apple plants.

Obviously, low levels of nitrogen significantly induced autophagic activity in apple plants, but the autophagosome formation seemed more frequent in *MdATG10*-overexpressing apple plants. In *Arabidopsis*, researches on the various *atg* mutants under suboptimal conditions demonstrated the important role of autophagy in nutrient recycling, especially under starvation conditions [23]. Here, we applied *MdATG10*-overexpressing apple plants to comprehensively analyze the effects of enhanced autophagy on the physiological changes and nitrogen utilization during apple growth under long-term low-nitrogen treatment. *MdATG10* overexpression could significantly increase the root–shoot ratio of apple plants, which promoted their uptake of nitrogen nutrients, thereby reducing the damaging effect of nitrogen deficiency on the whole plant growth. In addition, higher activities of nitrogen assimilation enzymes were observed in transgenic apple plants, together with the higher expression levels of nitrogen absorption genes. We believed that autophagy could improve the nitrogen utilization efficiency of apple plants by improving the absorption and assimilation of nitrogen. Therefore, the increased nitrogen utilization efficiency ensured the chloroplast activity in apple leaves, thus promoting their growth under nitrogen-deficient conditions. Besides the conserved degradation and circulation roles of autophagy, these findings provided insight into the autophagy-mediated morphological and physiological acclimation mechanisms of apple plants in response to nitrogen deficiency.

## 4. Materials and Methods

### 4.1. Plant Materials and Treatment

The *M. hupehensis* Rehd. apple plants treated with nitrogen depletion conditions were used to examine the expression pattern of *MdATG10* under low-nitrogen stress [50]. Tissues of GL-3 apple plants were cultured as described previously [51], and then sub-cultured every 4 weeks. After 30 days on the rooting media, GL-3 (WT) and three *MdATG10*-overexpressing apple lines (OE–1, OE–4, and OE–5) were planted in plastic pots (8 × 8 cm) filled with a substrate (Pinds Substrate)/roseite/perlite mixture (*v*:*v*:*v*, 3:1:1) and placed in a growth chamber (25/22 °C day/night, 120 µmol photons m^−2^ s^−1^, 14-h photoperiod). After acclimation for 20 days, transgenic and WT apple plants of similar size were transferred to a hydroponics system as described previously [52]. Plants of uniform size were selected for treatment after a 10-day preincubation. Each strain contained eighty plants was divided into two groups. The control group was cultured with 1/2 Hoagland nutrient solution (1.75 mM Ca(NO_3_)_2_, 2.5 mM KNO_3_, 0.5 mM KH_2_PO_4_, 1 mM MgSO_4_, 0.05 mM FeSO_4_, 0.05 mM EDTA-Na_2_, 23.13 μM H_3_BO_3_, 4.57 μM MnCl_2_, 0.38 μM ZnSO_4_, 0.16 μM CuSO_4_, 0.25 μM H_2_MoO_4._), and the nitrogen supply in the treatment group was decreased to 0.15 mM (1.75 mM CaCl_2_, 0.15 mM KNO_3_, 1.18 mM K_2_SO_4_, 0.5 mM KH_2_PO_4_, 1 mM MgSO_4_, 0.05 mM FeSO_4_, 0.05 mM EDTA-Na_2_, 23.13 μM H_3_BO_3_, 4.57 μM MnCl_2_, 0.38 μM ZnSO_4_, 0.16 μM CuSO_4_, 0.25 μM H_2_MoO_4_) [44,52]. They were cultivated under conditions of 23–25/16–18 °C Day/night, 160 µmol photons m^−2^ s^−1^, and a 14-h photoperiod, and the nutrient solution was changed every 5 days. After 14 and 28 days of this experiment, the third through sixth leaves from the apex of the stem (fully mature leaves) and the roots were sampled from all strains for damage analyses. For samples mentioned above, three biological replicates were prepared with three plantlets combined as one replicate. The samples were stored at −80 °C after being frozen quickly in liquid nitrogen.

### 4.2. Growth Measurements

Nine plants per strain were collected to measure the plant height and root length after the hydroponic treatment. Plant heights were measured from the base of the stem to the terminal bud of the main stem. After 0 and 28 days of the treatment, 15 plants per strain were collected and divided into root, stem, and leaf portions. The fresh and dry weights were measured as described previously [53]. Briefly, the three portions of plants were rinsed first with tap water, then with tap water containing 0.1 mol HCl, and finally with distilled water. After the fresh weight (FW) of each sample plant was recorded, the dry weight (DW) was obtained after the plants were fixed at 105 °C for 15 min and oven-dried at 75 °C for at least 72 h to a constant weight. The relative growth rate was calculated as follows: RGR = (ln DW_2_ − ln DW_1_)/ (T_2_ − T_1_), where DW_1_ is the plant DW on day 0 (T_1_) and DW_2_ is the plant DW on day 28 (T_2_). The root: shoot ratio was calculated as root DW/ (leave DW + stem DW).

### 4.3. Evaluation of Photosynthetic Characteristics and Chlorophyll Fluorescence

On days 0, 7, 14, 21, and 28 of the hydroponic experiment, the net photosynthesis rate (Pn) was monitored between 8:30 and 11:00 a.m. using a CIRAS-3 portable photosynthesis system (CIRAS, PP-Systems, Amesbury, MA, USA). All measurements were taken at 1000 μmol photons m^−2^ s^−1^ and a constant airflow rate of 500 μmol s^−1^. The concentration of cuvette CO_2_ was set at 400 ± 5 cm^3^ m^−3^. Data were collected from fully expanded, fully light-exposed leaves at the same position from six plants.

Chlorophyll fluorescence transients were measured on leaves at the same position from selected plants after 20 min of dark acclimation using the Open FluorCam FC 800-O, and *Fv/Fm* ratios were calculated with Fluorcam7 software (PSI, Brno, Czech Republic).

### 4.4. Measurements of Root Architecture and Root Activity

After 28 days of treatment, the roots of selected plants were cut off and rinsed with tap water and distilled water without damage. After using SNAPSCAN310 scanner (Seiko Epson Corp., Bangalore, India) to collect scanned images, the total root length, average diameter, root volume, surface area, and number of forks were measured using the WinRhizo image analysis system (V4.1 c; Regent Instruments, Quebec, QC, Canada).

To analyze root activity, fresh and white root samples of six plantlets were collected at the end of stress treatment. The root activity was determined through triphenyl tetrazolium chloride method using detection kits (Suzhou Comin Biotechnology Co. Ltd., Suzhou, China) following the manufacturer’s protocols.

### 4.5. Determination of Nitrogen Concentrations and Enzymatic Activities

The nitrogen concentrations were determined by the heating digestion method [53]. Briefly, after sieving with a 0.15 mm sifter, 0.1 g samples of roots, stems, and leaves were digested with 5 mL of concentrated sulfuric acid (H_2_SO_4_, AR, 98%) and hydrogen peroxide (H_2_O_2_, GR, ≥30%) at 375 °C. Deionized H_2_O was added to a volume of 100 mL. Nitrogen concentrations were obtained with an Auto Analyzer 3 (AA3) continuous-flow analyzer (SEAL Analytical, Norderstedt, Germany).

The activities of nitrate reductase (NR), nitrite reductase (NiR), glutamic acid synthase (GOGAT), and glutamine synthetase (GS) were determined using detection kits (Suzhou Comin Biotechnology Co. Ltd., Suzhou, China) following the manufacturer’s protocols at the end of stress treatment.

### 4.6. RNA Extraction and qRT-PCR Analysis

Total RNA extraction was carried out using a Wolact plant RNA isolation kit (Wolact, Hong Kong, China). Then, 1 μg of total RNA was used for cDNA synthesis using a RevertAid First-Strand cDNA Synthesis Kit (Thermo Scientific, Waltham, MA, USA). The qRT-PCR analysis was carried out with an SYBR Premix Ex Taq II Kit (TaKaRa, Dalian, China) using a LightCycler 96 quantitative instrument (Roche, Switzerland). Three biological replicates were conducted in each assay, and *Malate dehydrogenase* (*MdMDH*) transcription was used to normalize the levels of different genes [54]. The relative expression level of each gene was determined by the 2^−ΔΔCT^ method [55], and the specificity of each gene was determined using a dissociation curve analysis. All experiments were repeated with three biological replicates. The gene-specific primer sequences are shown in Supplemental Table.

### 4.7. Observation of Autophagosomes

On day 28 of the experiment, the fourth mature leaves from the apex of the stem were excised from selected plants and immediately cut into small pieces in 2.5% aldehyde solution before being placed in the dark for 12 h at 4 °C. After washing with 0.2 M PBS buffer (pH 7.4), the samples were fixed with 1% (*v*/*v*) osmium tetroxide for 2.5 h at room temperature, dehydrated in a graded ethanol series (30–100%; *v*/*v*), and embedded in Epon 812. The ultrathin sections (70 nm) were prepared on an ultramicrotome (Leica ULTRACUT, Wetzlar, Germany) and collected on Formvar-coated grids. The autophagosomes were observed under a Jeol-1230 transmission electron microscope (TEM, Hitachi, Tokyo, Japan) at an accelerating voltage of 80 kV.

### 4.8. Statistical Analysis

Experimental data were analyzed with SPSS 20.0 software. The statistical analysis was performed by one-way analysis of variance followed by Tukey’s multiple range test (*p* < 0.05), and values were presented as mean ± SEs (standard error) of at least three biological replicate samples.

## 5. Conclusions

As the *MdATG10* expression could be induced by a low nitrogen level both in the leaves and roots of apple plants, the *MdATG10*-overexpression apple plants were cultured in a hydroponic system containing different nitrogen levels for 28 days to investigate how autophagy functions in the physiological and growth state of apple plants under nitrogen starvation. The present study found that *MdATG10* overexpression induced the autophagic activity in apple plants, which led to a better root growth and a more efficient absorption and assimilation of the limited nitrogen sources under nitrogen starvation condition. This effectively absorbed nitrogen and nutrition also reduced the starvation damage to the chloroplasts in transgenic plants. It was concluded that besides the degradation role of autophagy, it could elevate the tolerance of apple plants to nitrogen deficiency by promoting the root–shoot ratio of plants and further reducing the starvation damage on the photosynthetic system.

## Figures and Tables

**Table 1:** Tissue fresh weights (FW, g) and dry weights (DW, g) measured from WT and *MdATG10*-OE plants after 28 d of nitrogen-deficiency treatment.

	Root FW (g plant^−1^)	Stem FW (g plant^−1^)	Leaf FW (g plant^−1^)	Total FW (g plant^−1^)	Root DW (g plant^−1^)	Stem DW (g plant^−1^)	Leaf DW (g plant^−1^)	Total DW (g plant^−1^)
WT–CK	1.063 ± 0.069 a	1.429 ± 0.043 a	3.675 ± 0.129 a	6.168 ± 0.229 a	0.206 ± 0.007 ab	0.815 ± 0.027 a	1.222 ± 0.093 a	2.244 ± 0.118 a
OE–1–CK	1.031 ± 0.109 a	1.408 ± 0.029 a	3.672 ± 0.075 a	6.110 ± 0.054 a	0.211 ± 0.006 ab	0.803 ± 0.008 a	1.223 ± 0.082 a	2.237 ± 0.076 a
OE–4–CK	1.081 ± 0.075 a	1.436 ± 0.036 a	3.634 ± 0.077 a	6.151 ± 0.112 a	0.207 ± 0.007 ab	0.805 ± 0.005 a	1.237 ± 0.113 a	2.249 ± 0.118 a
OE–5–CK	1.029 ± 0.071 a	1.425 ± 0.036 a	3.643 ± 0.058 a	6.089 ± 0.128 a	0.202 ±0.004 ab	0.803 ± 0.004 a	1.189 ± 0.062 a	2.196 ± 0.062 a
WT–ND	0.568 ± 0.076 b	0.726 ± 0.029 c	2.185 ± 0.069 c	3.476 ± 0.059 c	0.106 ± 0.009 d	0.256 ± 0.009 c	0.699 ± 0.045 c	1.062 ± 0.049 c
OE–1–ND	1.004 ± 0.037 a	1.024 ± 0.101 b	2.692 ± 0.065 b	4.717 ± 0.065 b	0.183 ± 0.005 c	0.299 ± 0.009 b	0.829 ± 0.012 bc	1.312 ± 0.026 b
OE–4–ND	1.122 ± 0.054 a	1.065 ± 0.131 b	2.739 ± 0.061 b	4.927 ± 0.207 b	0.197 ± 0.007 b	0.303 ± 0.008 b	0.866 ± 0.036 b	1.367 ± 0.026 b
OE–5–ND	1.097 ± 0.043 a	1.069 ± 0.132 b	2.938 ± 0.042 b	4.905 ± 0.131 b	0.201 ± 0.005 ab	0.303 ± 0.008 b	0.871 ± 0.026 b	1.375 ± 0.029 b

Note: CK, plants without any treatment; ND, plants with nitrogen deficiency treatment. All data are means ± SE of 9 plants. Values not followed by the same letter indicate significant differences between treatments, according to one-way ANOVA followed by Tukey’s multiple range test (*p* < 0.05).

**Table 2:** Root scanning analysis of WT and *MdATG10*-OE plants after 28 d of nitrogen deficiency treatment.

	Length (cm)	Surf Area (cm^2^)	Avg Diam (mm)	Root Volume (cm^3^)	Forks
**WT–CK**	1256.30 ± 42.30 ab	93.85 ± 4.16 a	0.24 ± 0.01 ab	0.63 ± 0.02 ab	26428 ± 3288.17 a
**OE–1–CK**	1288.40 ± 26.28a	93.88 ± 3.22 a	0.28 ± 0.02 ab	0.63 ± 0.01 a	25322 ± 2156.41 a
**OE–4–CK**	1296.20 ± 47.97 a	93.08 ± 1.28 a	0.28 ± 0.04 ab	0.65 ± 0.12 ab	26348 ± 2466.29 a
**OE–5–CK**	1284.70 ± 53.31 a	92.96 ± 2.45 a	0.26 ± 0.02 ab	0.65 ± 0.08 ab	23746 ± 2466.29 a
**WT–ND**	556.99 ± 26.72 d	42.21 ± 2.25 c	0.24 ± 0.01 b	0.26 ± 0.02 c	8692 ± 199.56 b
**OE–1–ND**	927.00 ± 17.36 c	78.33 ±8.01 b	0.28 ± 0.03 ab	0.55 ± 0.06 ab	20846 ± 1929.27 a
**OE–4–ND**	1160.50 ± 41.10 b	102.81 ± 2.89 a	0.25 ± 0.01 ab	0.66 ± 0.01 ab	22855 ± 1902.22 a
**OE–5–ND**	1161.60 ± 34.84 b	100.93 ± 3.28 a	0.30 ± 0.03 a	0.76 ± 0.04 a	22432 ± 1805.93 a

Note: CK, plants without any treatment; ND, plants with nitrogen deficiency treatment. All data are means ± SE of 5 plants. Values not followed by the same letter indicate significant differences between treatments, according to one-way ANOVA followed by Tukey’s multiple range test (*p* < 0.05).

## Data Availability

The data supporting the findings of this study are available within the article and its Supplementary Materials.

## Supplementary Materials

The following are available online at https://www.mdpi.com/article/10.3390/ijms22158085/s1.
