# Supplementary material for: Enhanced Autophagic Activity Improved the Root Growth and Nitrogen Utilization Ability of Apple Plants under Nitrogen Starvation
Source: Int J Mol Sci. 2021 Jul 28;22(15):8085. doi: 10.3390/ijms22158085 (PMC8348665; doi:10.3390/ijms22158085)
Supplement: Supplementary file 1 [file ijms-22-08085-s001.zip › ijms-1294351-supplementary.pdf]

Supplemental Table Primers used in this study

**Gene**	**Sequence (5'-3')**	**Purpose**
MDH	F: CGTGATTGGGTACTTGGAAC<br>R: TGGCAAGTGACTGGGAATGA	Reference gene used in real-time PCR
q*ATG3a*	F: AAGGGGGCGGAGATGGTTC<br>R: GCACTTAGAGACGAGGTTATCGC	Quantitative expression of *MdATG3a*
qATG3b	F: AGGGAGATGGTTTTGAAACAGA<br>R: ACTTAGAGACGAGGTTATCGC	Quantitative expression of *MdATG3b*
q*ATG4*	F: CACAATCTTCTTCAGGCTGGAA<br>R: CATCAAGGTCAGTTGCTTCTCT	Quantitative expression of *MdATG5*
qATG7a	F: GCGGATATGAGCAACCTTGGC<br>R: ATCAATAGGCGCAACGACATCA	Quantitative expression of *MdATG7a*
q*ATG8c*	F: GCGTTCAAGATGGAGCACCCTC<br>R: CAGCCCTTTCCACAACCACTGG	Quantitative expression of *MdATG8c*
q*ATG8f*	F: TCGTAGACAATGTCCTCCCAGC<br>R: CCAAATGTGTTCTCGCCACTGT	Quantitative expression of *MdATG8f*
q*ATG8i*	F: GCAGCAGGCTTCACTTGACTCC<br>R: GGAATCCATGCGACTGGCTGTT	Quantitative expression of *MdATG8i*
q*ATG9*	F: ACTTCATGCGTCAGCCTTCAGA<br>R: CGTTCCTCCAATCCAACCGTTG	Quantitative expression of *MdATG9*
qATG12	F: ACAGTGCATTCTCGCCAAACCC<br>R: CCCCATGCCATGGAGCAAGC	Quantitative expression of *MdATG12*
q*NRT1.1*	F: CTGGCTGGTCCCACAGTTCTT<br>R: CTTCATTCCTTTCGGGCACTC	Quantitative expression of *MdNRT1.1*
qNRT2.4	F: CAGAAGGTGAACCCGGAAG<br>R: CAAGTGGAACGTCCTCATGTG	Quantitative expression of *MdNRT2.4*
q*NRT2.5*	F: TTGTGGTCCATCTAAGAACAAGGC<br>R: TCATCAGAGGGTCGGGTAACAG	Quantitative expression of *MdNRT2.5*
q*NRT2.7*	F: TCTCCAGGCAGACGAGCATT<br>R: GGAGCAAGTGATACTGGTTTGTTTC	Quantitative expression of *MdNRT2.7*
q*AMT1.1*	F: ATCCGACGCCAACTACACTTCTAC<br>R: CGATTTGGTCACAGATGAAGTGAG	Quantitative expression of *MdAMT1.1*
q*AMT1.2*	F: CTGGCTGGTCCCACAGTTCTT<br>R: CTTCATTCCTTTCGGGCACTC	Quantitative expression of *MdAMT1.2*
q*AMT1.6*	F: GTAACTTATTGTTCACCTCTGGGGC<br>R: CGTTCCTGAACGCGTCGAAT	Quantitative expression of *MdAMT1.6*
q*AMT2.1*	F: GTGACGATGGATCGATTGAGACTC<br>R: CCCGCTAACAAAATAAGAGTAATAGCT	Quantitative expression of *MdAMT2.1*
